# Assessing Facilitator Fidelity to Principles of Public Deliberation: Tutorial

**DOI:** 10.2196/51202

**Published:** 2023-12-13

**Authors:** Claire Draucker, Andrés Carrión, Mary A Ott, Amelia Knopf

**Affiliations:** 1 School of Nursing Indiana University Indianapolis, IN United States; 2 Department of Pediatrics School of Medicine Indiana University Indianapolis, IN United States

**Keywords:** public deliberation, deliberative democracy, bioethics, engagement, theory, process, ethical conflict, ethical, ethics, coding, evaluation, tutorial, biomedical, HIV, HIV prevention, HIV research

## Abstract

Public deliberation, or deliberative democracy, is a method used to elicit informed perspectives and justifiable solutions to ethically fraught or contentious issues that affect multiple stakeholder groups with conflicting interests. Deliberative events bring together stakeholders (deliberants) who are provided with empirical evidence on the central issue or concern and then asked to discuss the evidence, consider the issue from a societal perspective, and collectively work toward a justifiable resolution. There is increasing interest in this method, which warrants clear guidance for evaluating the quality of its use in research. Most of the existing literature on measuring deliberation quality emphasizes the quality of deliberants’ inputs (eg, engagement and evidence of compromise) during deliberative sessions. Fewer researchers have framed quality in terms of facilitator inputs, and these researchers tend to examine inputs that are consistent with generic group processes. The theory, process, and purpose of public deliberation, however, are distinct from those of focus groups or other group-based discussions and warrant a mechanism for measuring quality in terms of facilitator fidelity to the principles and processes of deliberative democracy. In our public deliberation on ethical conflicts in minor consent for biomedical HIV prevention research, we assessed facilitator fidelity to these principles and processes because we believe that such assessments serve as a component of a comprehensive evaluation of overall deliberation quality. We examined verbatim facilitator remarks in the deliberation transcripts and determined whether they aligned with the 6 principles of public deliberation: equal participation, respect for the opinions of others, adoption of a societal perspective, reasoned justification of ideas, expression of diverse opinions, and compromise or movement toward consensus. In this tutorial, we describe the development of a blueprint to guide researchers in assessing facilitator fidelity, share 3 templates that will assist them in the task, and describe the results of our assessment of facilitator fidelity in 1 of the 4 sites in which we conducted deliberations.

## Introduction

Public deliberation is a process used to obtain informed citizen input for the development and implementation of public policies that are novel, contested, or divisive [1,2]. Diverse members of the public are recruited to represent competing interests and gathered to thoughtfully consider policy questions [3]. The goals of public deliberation, also referred to as consensus conferences, citizen juries, and deliberative democracy, are to provide a forum in which citizens can shape strategic decision-making and inform policies that affect them [1,2,4].

The design, methods, and models of public deliberations vary but typically consist of 3 components [1,2,4,5]. First, experts provide deliberants with factual, balanced, and focused information related to a public policy decision. Second, deliberants discuss their values, experiences, and viewpoints related to the decision and weigh competing options. Third, deliberants identify collective values, commonalities in perspectives, and acceptable trade-offs to reach an informed and reasoned consensus opinion.

Public deliberation is particularly suited for controversial policy decisions in health care and bioethics [6]. However, with the growing use of public deliberations, there is a need to assess their quality [1,4,7]. Researchers recommend a variety of approaches to evaluating the quality of deliberative events.

Some researchers evaluate quality by focusing on deliberants’ experiences and the extent to which these experiences are consistent with deliberative goals. For example, the Public and Patient Engagement Evaluation Tool [8] was developed to assess several dimensions of the deliberative experience and includes items to measure communication and support (*I had a clear understanding of the purpose of the deliberation),* the sharing of perspectives (*I was able to express my views freely),* and the impacts of participation (*I think that the deliberation achieved its objectives).* Some researchers consider change in deliberants’ opinions and preferences indicative of a successful deliberation, although others caution against relying on this metric because change may be due to power differentials, social desirability, or other structural dynamics that deliberations are designed to mitigate [9]. Some researchers include inclusivity as a criterion for quality and systematically assess the extent to which all stakeholder groups, including those who might be marginalized, are recruited and retained for deliberation [1].

Several researchers have proposed a multidimensional approach to evaluate deliberation quality. For example, Goold et al [7] proposed an analytic framework to examine the quality of deliberations in the following 3 domains: structure (ie, how the deliberation is organized), process (ie, how the deliberation transpires), and outcomes (eg, the nature of consensus opinions reached by deliberants). Similarly, Scott et al [10] merged several published frameworks into an overarching structure to evaluate quality that included 4 deliberation elements (eg, jurors’ preferences and values, engagement with each other, referencing expert information, and enrichment of deliberation) and 4 recommendation elements (eg, clear and identifiable recommendations, recommendations that address the deliberation question, justification of recommendations, and adoption of a societal perspective).

Multiple data sources, such as deliberant surveys, follow-up interviews with deliberants and stakeholders, and deliberation transcripts, are typically used to conduct quality assessments. These data sources are analyzed using both qualitative and quantitative approaches [3,10]. For example, some process elements are measured using instruments such as the Public and Patient Engagement Evaluation Tool [8], but others require qualitative scrutiny of deliberative transcripts to examine interactive processes that occur during deliberative events.

In many of these approaches, the role of facilitators, including their fidelity to the principles of deliberation, has not been considered. One exception is the discussion of facilitation quality by De Vries et al [4]. They counted how many words facilitators spoke, examined the contributions of the outliers (ie, very talkative and very quiet facilitators), and provided examples of good facilitation (eg, encouraging reluctant deliberants and encouraging deliberants to give reasons for their views) and less optimal facilitation (eg, allowing discussions to go on too long and inserting personal opinions). However, little guidance is available on how to comprehensively assess facilitator fidelity according to a set of principles of deliberative democracy, which is the gap we intended to fill. We believed that an optimal approach to evaluating facilitator fidelity is to examine verbatim facilitator remarks in the context of deliberative discussions.

Therefore, we developed a blueprint to guide the process of assessing facilitator fidelity during our own deliberations. The blueprint includes coding templates and step-by-step analytic procedures using which each relevant facilitator remark is rated based on the extent to which it upholds the basic principles of public deliberation. Fidelity assessments based on such a blueprint can provide metrics of deliberation fidelity, inform facilitator training, and provide a better understanding of how facilitator input advances or impedes the goals of public deliberation. The purpose of this tutorial, therefore, is to present the blueprint we developed to systematically assess facilitator remarks made during a public deliberation about the acceptability of allowing minor consent for biomedical HIV prevention research. We briefly describe the deliberation, present the coding templates, list the analytic procedures, describe the assessment results from 1 deliberation site, and provide examples of how the assessment results were used to optimize future deliberations.

## Public Deliberation: Improving Consent Processes for Engaging Underrepresented Populations in Clinical Research

We provide a brief description of our deliberation research to provide the context for the development of the blueprint for assessing facilitator fidelity. Additional details about the sample, deliberation procedures, and deliberation outcomes are presented elsewhere [11,12].

The public health problem that was the focus of our deliberation is underrepresentation or exclusion of minor adolescents from clinical trials of biomedical HIV prevention methods, which subsequently limits their access to effective HIV prevention strategies and contributes to a disproportionate burden of HIV incidence among this group [13]. Engaging minor adolescents in biomedical HIV prevention trials is difficult, in part because of the barriers that arise in consent processes [14,15]. Parental consent and minor assent are typically required for minor participation in biomedical clinical trials to guard against research-induced harms [16,17]. However, this model can be harmful to some minors who would be required to reveal previously undisclosed stigmatized behaviors and identities to their parents, thereby risking rejection or punishment [18]. These risks create the need for improved consent methods, including the consideration of flexible approaches to consent and models that include minor self-consent [19].

However, calls for new consent models for HIV prevention research for minor adolescents, especially those that would allow minor self-consent, have raised ethical conflicts due to the competing interests and duties of those who hold a stake in this research [20]. Stakeholders include adolescents at risk for HIV, parents of minor adolescents, community leaders, investigators and their institutions, and institutional review boards. The strongly held and often conflicting interests of stakeholders in adolescent HIV prevention have made it difficult to resolve the ethical conflicts that contribute to the underrepresentation of minors in HIV clinical trials. Similar barriers to consent complicate the enrollment of other populations in clinical trials [21]. Therefore, the goal of our public deliberation was to use the issue of minor consent for biomedical HIV prevention research to test public deliberation as a method for improving consent processes for engaging underrepresented populations in clinical research.

The public deliberations were convened over a web-based video platform and engaged participants from 4 cities selected for their geographical and cultural diversity and high rates of HIV and other sexually transmitted infections: Tampa, Florida (site 1); Baltimore, Maryland (site 2); Denver, Colorado (site 3); and Chicago, Illinois (site 4). The deliberants included youths, parents, caregivers, and community members who worked with youths. With input from youth advisory boards and content experts, deliberation materials were developed, including a participant website outlining key elements of the deliberation, videos and other educational content related to deliberation topics (eg, regulatory processes, HIV infection, clinical trial procedures, and disclosure risks among youths with marginalized identities, especially lesbian, gay, bisexual, and transgender youth), facilitation guides for facilitator training, and key questions to be addressed in the deliberation. The materials were reviewed by external advisers with expertise in deliberation.

For the purpose of this tutorial, we focused exclusively on site 1. At site 1, a total of 57 individuals were screened, and 60% (n=34) of them were eligible to participate. Among these 34 individuals, 20 (59%) consented to enrollment, of whom 15 (75%) participated in the deliberation. The age of the deliberants ranged from 14 to 60 years; 4 (27%) were teens, 2 (13%) were young adults (aged 20-24 years), and 9 (60%) were adults. Of the 15 deliberants, 12 (80%) were women, 1 (7%) was a man, and 2 (13%) identified as both women and gender nonbinary. Overall, 7 (47%) deliberants identified as White, 5 (33%) as Black or African American, 1 (7%) as Asian, 1 (7%) as multiracial, and 1 (7%) did not specify their racial identity. Moreover, 2 (13%) deliberants identified as Hispanic or Latinx.

Deliberations were conducted over a web-based video platform for 2 hours 1 day per week for 4 weeks. Thus, there were 4 sessions. Each session had a plenary discussion; in sessions 1 and 2 deliberants were divided into 2 smaller breakout groups to allow time for rapport building and dialogue. A total of 11 research team members were involved in the deliberations. The sessions included 4 stakeholder presentations, expert testimonies, and large (plenary) and small (breakout group) sessions. The presentations and testimonies were provided by research team members (n=2) and expert stakeholders (eg, a physician who conducts HIV clinical trials, institutional review board administrator; n=2) and addressed the following topics: research with adolescents, adolescent medicine, ethics, trial design, and local context. The deliberations were led by plenary facilitators (n=2) who were experts in public deliberations and breakout group facilitators (n=2) who were trained graduate students. Most of the remarks coded for fidelity were made by the plenary and breakout group facilitators, and other team members made less frequent intermittent remarks during the deliberative discussions, which were also coded. Therefore, hereinafter, all these persons are referred to as facilitators. The sessions were recorded and transcribed. The transcribed recordings of all the deliberation sessions at site 1 served as the data source for this report.

## Development of a Blueprint for Assessing Facilitator Fidelity

### Overview

To develop a blueprint for assessing facilitator fidelity, we drew from the work of De Vries et al [4]. On the basis of their review of the literature, they identified 4 core principles of quality deliberations: equal participation (ie, equal contributions among deliberants), respect for the opinions of others (ie, positive or amicable interactions even during disagreements), adoption of a societal perspective (ie, focus on the common or civic good rather than self-interests), and reasoned justification of ideas (ie, opinions supported with factual information or rational thinking). We added 2 additional principles based on the goals of our deliberation: expression of diverse opinions (eg, articulation of differing or divergent views) and compromise or movement toward consensus (eg, work toward finding a common ground).

We reasoned that a logical indication of facilitator fidelity was the extent to which facilitator remarks made during deliberative discussions were aligned with these principles. Our assumption was that remarks consistent with a core principle would serve to encourage or invite deliberants to engage in discussions that uphold the principle, whereas remarks not aligned with a principle might thwart or divert such discussions. To assess alignment, we assigned persons who were not facilitators of the deliberations (hereafter referred to as raters) to code each pertinent facilitator remark to a core principle and indicate whether the remark was consistent or inconsistent with the principle.

### Templates

To expedite the coding process, we developed 3 templates to facilitate the process. Template 1 displays the coding rules, template 2 is a table for organizing and displaying the codes, and template 3 is a table for displaying the final code counts.

#### Template 1: Coding Rules

This template displays the 6 core principles, coding rules for guiding the coding of facilitator remarks and their identification as consistent (eg, encourages equal participation) or inconsistent (eg, discourages equal participation) with each principle, and examples of coded text. If a remark is coded as inconsistent, this does not indicate that the remark was necessarily detrimental to the deliberation but rather that it varied from the essence of a principle in some way. Because we aimed to evaluate fidelity to the deliberation process specifically, the rules specify that remarks reflecting general group facilitation techniques, such as those made in a traditional focus, discussion, or therapy groups, are not rated. For example, if a facilitator asked a participant to clarify a comment, the remark would be considered a generic group facilitation technique and not coded. However, if the facilitator asked for the reason for a deliberant’s opinion, the remark would be coded reasoned justification of ideas. In addition, prepared stakeholder presentations and expert testimonies and procedural comments (eg, “turn on your camera” and “our time is almost up”) were not coded. The coding rules were modified iteratively through several initial coding efforts. An example of the coding rules template is presented in Table S1 in Multimedia Appendix 1.

#### Template 2: Code Display Table

This table lists the core principles on the row headers and facilitator roles (ie, plenary facilitators, breakout group facilitators, research team members, expert stakeholders, and site staff) below each core principle. The plenary discussion and breakout sessions are listed on the column headers; of note, breakout groups are named by session and small group number (eg, Breakout 1.1 refers to session 1, small group 1). The table provides cells for raters to insert the transcript line numbers of facilitator remarks that were assigned particular codes. For example, on lines 74-79 of the transcript of the plenary discussion in session 1, plenary facilitator 1 made remarks that were consistent with the principle of equal participation. Thus, lines 74-79 were entered in the cell that corresponds to the principle of equal participation and the plenary discussion, session 1. The table provides an overview of the distribution of the coded remarks across sessions and facilitators for each site as well as a mechanism to cross-reference the codes with the original transcript data. An example of the code display table (template 2), completed for site 1, is presented in Table S2 in Multimedia Appendix 2.

#### Template 3: Code Summary Table

This table lists the core principles in the row headers and the plenary and breakout group sessions in the column headers. Raters sum the remarks made by all facilitators and place these totals in the appropriate cells. The raters then sum all remarks coded to each principle for the entire deliberation at each site. An example of the code summary table (template 3), completed for site 1, is presented in Tables 1 and 2.

**Table 1:** Code summary table example.

			Session 1 plenary remarks, n		Session 1 breakout group 1 remarks, n		Session 1 breakout group 2 remarks, n		Session 2 plenary remarks, n		Session 2 breakout group 1 remarks, n		Session 2 breakout group 2 remarks, n		Session 3 remarks, n		Session 4 remarks, n		Total remarks, n
**Remarks consistent with core principles (n=93)**																			
	EP^a^	1		1		0		0		0		2		3		2		9	
	RO^b^	4		0		0		1		1		2		0		0		8	
	ED^c^	3		1		1		3		11		8		5		4		36	
	SP^d^	1		0		1		0		1		1		0		0		4	
	RJ^e^	0		0		1		0		1		1		2		2		7	
	CC^f^	10		1		0		5		3		0		1		9		29	
**Remarks inconsistent with core principles (n=18)**																			
	EP	0		0		0		0		0		0		1		1		2	
	RO	0		0		0		0		0		0		0		0		0	
	ED	0		0		0		0		2		1		0		2		5	
	SP	0		1		1		0		0		0		0		0		2	
	RJ	0		0		0		0		0		0		0		0		0	
	CC	0		0		0		3		1		0		0		5		9	

^a^EP: equal participation.

^b^RO: respect for others.

^c^ED: expression of diverse opinions.

^d^SP: adoption of a societal perspective.

^e^RJ: reasoned justification of ideas.

^f^CC: compromise or movement toward consensus.

**Table 2:** Abbreviated summary.

Core principle	Consistent remarks, n	Inconsistent remarks, n	Total, n
EP^a^	9	2	11
RO^b^	8	0	8
ED^c^	36	5	41
SP^d^	4	2	6
RJ^e^	7	0	7
CC^f^	29	9	38

^a^EP: equal participation.

^b^RO: respect for others.

^c^ED: expression of diverse opinions.

^d^SP: adoption of a societal perspective.

^e^RJ: reasoned justification of ideas.

^f^CC: compromise or movement toward consensus.

### Step-by-Step Analytic Procedures

The blueprint outlines 8 analytic steps to systematically assess facilitator fidelity. This process is consistent with directed content analysis (Hsieh and Shannon [22]) in which narrative data are coded to a preexisting framework. The steps are as follows: (1) recorded deliberations in all sessions from each site are transcribed, and transcripts are prepared and stored for analysis; (2) transcripts are read by 2 raters in their entirety to obtain a sense of the overall focus and flow of the deliberation sessions; (3) the raters highlight facilitator remarks on the transcripts that reflect a core principle of deliberation (as described earlier); (4) the 2 raters independently code these remarks according to the coding rules template (Table S1 in Multimedia Appendix 1) using code abbreviations (eg, RO=respect for others’ opinions) or preassigned font colors (eg, adoption of a societal perspective=green); (5) the raters compare their codes and resolve discrepancies through discussion, consensus, and a review of the coding rules or re-examination of transcript data; (6) the transcript line numbers of all agreed upon coded remarks are displayed in the appropriate cell of the code display table (Table S2 in Multimedia Appendix 2); (7) the codes are summarized across facilitators and sessions, and the totals are placed in the code summary table (Tables 1 and 2); and (8) a narrative summary of facilitator fidelity at each site is prepared. A narrative summary of the deliberation at site 1 is presented in the subsequent section as an example.

## Findings From Deliberation Site 1

### Overview

In total, 111 facilitator remarks pertinent to a core principle were highlighted or extracted and coded. The facilitator remarks coded to each core principle are summarized in the subsequent subsections. The core principles are discussed in the order of the number of remarks coded to them.

### Expression of Diverse Opinions

A total of 41 remarks were coded to the expression of diverse opinions principle.

#### Consistent With the Principle

Of the 41 remarks, 36 (88%) were coded as consistent with the expression of diverse opinions principle. These remarks were dispersed throughout the deliberation sessions and made by a variety of facilitators, although they were most prevalent in the 2 breakout sessions of session 2. For the most part, these remarks encouraged deliberants to share perspectives that had not been previously discussed. For example, a plenary facilitator said, “I’ve heard a bunch of people saying that they don’t necessarily think that this [focusing on consent with cisgender youths] would really drive their decision-making. Is there anybody for whom it would?” (session 3). In another instance, a breakout group facilitator said, “and I wonder if people maybe had any additional perspectives that they think inform some of those values and that they might want to bring in here” (session 2, breakout group 2). In some remarks, facilitators encouraged deliberants to consider diverse opinions of groups or people not represented in the deliberation. A breakout group facilitator said, “are there any other perspectives that you think we’re missing from this conversation, or would be, I mean,... really helpful in hearing about in this context?” (session 2, breakout group 2).

#### Inconsistent With the Principle

Of the 41 remarks, 5 (12%) were coded as related to but inconsistent with the expression of diverse opinions principle. The remarks included expressions of personal opinions rather than requests for diverse opinions from deliberants. We chose to code these remarks as expression of diverse opinions (inconsistent) because deliberation facilitators are typically dissuaded from expressing their own opinions, as they can squelch dissenting or challenging views of deliberants [3,4]. Of these 5 remarks, 3 (60%) were made by expert stakeholders or site staff. In one instance, an expert stakeholder offered an opinion that researchers are not respectful of youths and do not consider the importance of their accessibility to research (session 2, breakout group 1). By emphasizing their own opinion regarding the importance of access, the expert stakeholder may have dissuaded deliberants from expressing conflicting perspectives. Other remarks were inconsistent with the expression of diverse opinions principle because the facilitator asked for opinions to support what had already been said, rather than inviting competing ideas.

### Compromise or Movement Toward Consensus

A total of 38 remarks were coded to the compromise or movement toward consensus principle.

#### Consistent With the Principle

Of the 38 remarks, 29 (76%) were coded as consistent with the compromise or movement toward consensus principle. Most of these remarks were made in session 1 plenary (10/29, 34%) and session 4 plenary (9/29, 31%), and most were made by a plenary facilitator. In session 1 plenary, these remarks were typically introductory and introduced the concept of compromise or consensus as important to the deliberative process. For example, the session 1 plenary facilitator said, “it [the deliberation] really is as much of a collaborative process where we’re creating something or identifying where we do have common ground or perhaps even creating some new common ground together” (session 1, plenary). In session 4 plenary, the remarks instructed the group to write a consensus summary statement. A plenary facilitator said the following:

So, in just a couple of minutes, I do want to move us on to actually trying to come up—we have gotten a lot of really, really great ideas out here, a lot of kind of if-then statements, a lot of different possibilities for how this could happen. And we’d like to try to synthesize those and summarize those into like a general, short like three to four sentences kind of statement of what we think the most important principles are and under what circumstance.

session 4, plenary

#### Inconsistent With the Principle

Of the 38 remarks, 9 (24%) were coded as related to but inconsistent with the compromise or movement toward consensus principle. Most of these remarks (5/9, 56%) occurred in the latter part of session 4 and were made by a variety of facilitators. The facilitators provided a summary of what they perceived the consensus of the group to be rather than inviting deliberants to articulate the consensus of the group as they perceived it. For example, a plenary facilitator said the following:

So, if I’m hearing you right, we’re saying that at the low end of low risk, there might not be parental consent needed at all. And at the super high end of risk, there would absolutely need to be parental consent. But in the middle, if it’s been tested on adults before, then there might be other options that would be possible—that would be potentially safe for a person—for a teen. And we’ve talked about some of what those might be. They might be an ombudsman. They might be an advocate that is well known to the teen. Or it might be a pre—an advance community consultation.

session 4, plenary

### Equal Participation

A total of 11 remarks were coded to the equal participation principle.

#### Consistent With the Principle

Of the 11 remarks, 9 (82%) were coded as consistent with the equal participation principle. These remarks were dispersed throughout the sessions and made by a variety of facilitators. The remarks drew out deliberants who had not spoken or who had not been active in the sessions. For example, a plenary facilitator said, “comments from others, anybody, especially folks that we haven’t heard from yet. What sticks out to you about this study, any particular concerns?” (session 3, plenary).

#### Inconsistent With the Principle

Of the 11 remarks, 2 (18%) were coded as related to but inconsistent with the equal participation principle. These remarks called on people who had already spoken often instead of those who had not spoken. A plenary facilitator said, “so, before we do that, I thought we just give one last comment to the one that—to the person that kicked this part of the discussion off. Deliberant X, would you like to say anything else?” (session 4, plenary).

### Respect for Others

A total of 8 remarks were coded to the respect for others principle.

#### Consistent With the Principle

All 8 (100%) remarks were coded as consistent with the respect for others principle. Several of these remarks (3/8, 38%) were made in the session 1 plenary by a plenary facilitator and were made to introduce the principle as an important aspect of the deliberation. For example, a plenary facilitator said, “so these are the basic ground rules for our discussion. Basically a matter of being respectful and collaborative...” (session 1, plenary). Other remarks conveyed approval when deliberants showed respect for other deliberants. For example, later in a breakout group, the breakout facilitator said, “thank you for sharing that, and for listening to what Deliberant X had to say and being able to build on, I think that’s really important. And, you know, I think that’s a, you know, a great example of being able to actively listen...” (session 2, breakout group 2).

#### Inconsistent With the Principle

No remarks were coded as related to but inconsistent with the respect for others principle.

### Reasoned Justification of Ideas

A total of 7 remarks were coded to the reasoned justification of ideas principle.

#### Consistent With the Principle

All 7 (100%) remarks were coded as consistent with the reasoned justification of ideas principle. These remarks were dispersed throughout the sessions and made by several facilitators. These remarks encouraged deliberants to use the information they had been presented or describe the rationale for their decisions. For example, a plenary facilitator said, “We want to give reasons for—in sentences we are writing, we want to also say why we think this, not only that we think it should be done.” (session 4, plenary).

#### Inconsistent With the Principle

No remarks were coded as related to but inconsistent with the reasoned justification of ideas principle.

### Adoption of a Societal Perspective

A total of 6 remarks were coded to the adoption of a societal perspective principle.

#### Consistent With the Principle

Of the 6 remarks, 4 (67%) were coded as consistent with the adoption of a societal perspective principle. These remarks were dispersed throughout the sessions and made by several facilitators. The remarks encouraged deliberants to consider the good of society or civic benefit or showed approval when they did so. For example, a research team member said, “was it—I thought I saw you sort of resonating with Deliberant X’s comment about individuality versus collectivism. And like thinking about whether something benefits me versus the community and sort of which way” (session 2, breakout group 1).

#### Inconsistent With the Principle

Of the 6 remarks, 2 (33%) were coded as related to but inconsistent with the adoption of a societal perspective principle. These remarks encouraged deliberants to focus on how the issue affected them rather than society as a whole. For example, a breakout group facilitator said, “and so we’re going to be asking—or I’ll be asking you a little bit about how this—the subject at hand with youth HIV, how it affects you, your family, and your communities, as well as maybe any stories or experiences you want to share” (session 1, breakout group 1).

## Using the Findings to Optimize Subsequent Deliberations

The overall goal of a facilitator fidelity assessment is to provide an indication of the extent to which facilitator input contributed to a high-quality deliberation. The facilitator fidelity assessment at site 1 allowed us to conclude that a high degree of fidelity to deliberation principles had occurred. Of the 111 remarks determined to be pertinent to a core deliberation principle, 93 (84%) were considered to be consistent with the principle.

We also used the information obtained via the assessment to iteratively fine-tune facilitator input for subsequent deliberations. We recognized that the number of remarks coded to each principle alone did not necessarily suggest a need to modify facilitator input. For example, we concluded that remarks coded to some core principles (eg, respect for others) were not prevalent because deliberants were already engaging in discussions reflecting the principle. Conversely, we concluded that remarks coded to some other core principles (eg, adoption of a societal perspective) were also not prevalent, but more such remarks were needed to meet the aims of the deliberation (eg, a focus on the civic good).

To inform future deliberations, a research team meeting was held, in which the raters presented the findings and conclusions to the research team. The team then decided what changes should be implemented based on this information. The conclusions, team discussion, and changes made to subsequent deliberations are presented in Table 3.

**Table 3:** Implementing facilitator fidelity assessment findings.

Conclusions from the fidelity assessment	Team discussion	Changes made to subsequent deliberations
The core principles least frequently reflected in the site 1 deliberations were reasoned justification of ideas and adoption of a societal perspective.	As reasoned justification of ideas and adoption of a societal perspective are key components of our deliberation goals, strengthening facilitator remarks that invite or encourage discussion related to these principles is needed.	The team developed exemplar responses that would align with the principles of reasoned justification of ideas (eg, what information did you hear today that influenced your opinion?) and adoption of a societal perspective (eg, in this deliberation, we will ask you to consider what would be best for society as a whole). The exemplars were placed on slides and provided as suggestions at a subsequent facilitator training session. The training also included a discussion of the meaning of adopting a societal perspective, as there seemed to be some confusion about this principle.
The core principle of expression of diverse opinions was well represented in the deliberation. These remarks involved asking deliberants whether they had opinions different than what had been discussed or asking them whether there were groups not represented in the deliberations who might have different opinions. Remarks inconsistent with this principle occurred in a few instances when facilitators expressed their own opinions.	Although few remarks conveyed the personal opinions of facilitators, the elimination of all statements of personal opinions is desired.	Facilitator training emphasized that expressing personal opinions should always be avoided during the deliberative discussion. The training also emphasized that potential power differentials between facilitators and deliberants might serve to quiet deliberant voices that may diverge from the opinions of facilitators if expressed.
Remarks related to the core principle of equal participation were mainly consistent with the principle. In only a couple of instances did a facilitator call on a person who had already spoken frequently.	More strategies to draw out quieter members, especially youth deliberants, are needed.	The team developed a variety of exemplar facilitator responses that could be used to encourage quieter members to speak and to respectfully request that dominant members allow and encourage the participation of more reticent deliberants (eg, it is important that we hear from as many deliberants as we can in our group tonight. We invite those who have not yet shared their thoughts on xxxx to do so). These responses were presented in facilitator training. The team also determined that it was advisable to directly engage quieter members if done in an inviting and nonthreatening manner (eg, XXXX, we would love to hear your thoughts on xxxxx. I believe your perspective would be important here).
Compromise or movement toward consensus was the core principle with which the largest number of remarks were inconsistent. Most of these remarks were those in which facilitators pronounced what consensus they believed the group had reached rather than asking deliberants to articulate what they viewed as consensus. Most of these remarks happened in the latter part of session 4 and might have been aimed at getting a consensus statement before the deliberation ended.	Strategies to ensure that the consensus opinions are attributable to deliberants would improve the deliberation process.	Strategies were developed to encourage deliberants to come to a consensus and to provide opportunities for deliberants to articulate their consensus opinions. Specifically, in each breakout session, deliberants were asked to summarize the consensus, and a volunteer was sought to report back to the larger group. Moreover, because the tendency for facilitators to provide their interpretation of the consensus reached by the group seemed to stem from “running out of time” at the end of sessions, plans to allow more time to come to and articulate consensus opinions were built into the deliberation run of show.

## Discussion

The blueprint presented here can provide guidance for researchers using public deliberation who wish to systematically assess the extent to which facilitator interactions with deliberants demonstrate fidelity to the core principles of public deliberation. By developing a preestablished coding schema and systematically coding and counting each facilitator remark specific to deliberative processes, we were able to ascertain the extent to which facilitator responses aligned with the principles of public deliberation and to identify instances when they did not.

Some limitations to this approach are noted. We recognize that counting the number of facilitator remarks related to each principle and determining whether the remarks were consistent or inconsistent with the principle does not reflect a definitive quantitative metric to assess fidelity. Rather, facilitator remarks need to be considered in the context in which they are offered, and the counts should be interpreted by the research team in light of the overall goals of the deliberation. For example, facilitator remarks that focus on inquiring about deliberants’ personal experiences (coded as adoption of a societal perspective—inconsistent) may be facilitative of rapport building early in the deliberation but might hinder discussions focused on the civic good later in the deliberation. Thus, fidelity counts serve as guideposts that point to areas that might need to be addressed to improve the quality of deliberations and lead to robust, reasoned, and relevant consensus opinions.

Another limitation is that the blueprint is based on one set of deliberative principles, as outlined by De Vries et al [4], whereas some deliberations might be guided by other principles. For example, Trinidad et al [23] conducted a deliberation with American Indian and Alaskan Native tribal leaders to explore the use of precision medicine in their communities. Although their deliberation was based on many of the principles discussed by De Vries et al [4], Trinidad et al [23] argued that the principles of reasoning, debate, and coming to consensus may discount some voices, and other principles such as valuing varied speech forms, emotional expression, and disagreement may better promote egalitarianism in deliberations. Thus, our blueprint would need to be modified to assess facilitator fidelity in the study by Trinidad et al [23]. We suggest that this could be done by operationalizing the applicable set of principles and developing relevant coding rules so that the blueprint could be used with one’s study.

The facilitator fidelity assessment conducted for site 1 is being replicated for the other 3 sites for an overall facilitator fidelity assessment. We are currently creating a master table that displays all fidelity counts for all facilitators for all sessions across the 4 sites. Such a display will allow us to examine the patterns of facilitator responses, determine whether these patterns change over the course of the deliberations, compare responses between facilitators across sites, and determine whether team training, as described in this tutorial, results in better alignment between deliberative principles and facilitator remarks. We also intend to develop analytic strategies to examine the nuances of interactions between facilitators and deliberants. These strategies will allow us to ascertain whether certain types of facilitator remarks trigger proximal deliberant responses, influence the deliberative tenor of the group over time, or affect deliberation outcomes.

The facilitator fidelity assessments will then be incorporated into a comprehensive assessment of quality. For this assessment, we will use a variety of analytic approaches and data sources to examine the deliberation structure, processes, and outcomes guided by the evaluation models of Goold et al [7] and others [8,9].

## Conclusions

Public deliberation offers a promising approach to obtaining citizen input on public policy decisions. To ensure the credibility of this approach, researchers and other stakeholders need to ensure that deliberations produce reasoned and informed consensus opinions and, therefore, need to continually assess and improve the quality of the deliberations. These assessments should include a close examination of the fidelity of facilitator remarks to the core principles of public deliberation. Our blueprint, which includes a coding schema, tables to expedite coding, and a delineation of analytic procedures, can be used to expedite fidelity assessments.

## Appendix

Multimedia Appendix 1 Coding rules table example.

Multimedia Appendix 2 Code display table example.
