# Supplementary material for: Assessing Facilitator Fidelity to Principles of Public Deliberation: Tutorial
Source: JMIR Form Res. 2023 Dec 13;7:e51202. doi: 10.2196/51202 (PMC10753414; doi:10.2196/51202)
Supplement: Multimedia Appendix 1 [file formative_v7i1e51202_app1.docx]

**Table S1.** Coding rules table example.

| General coding | | Do code | Do not code | Examples |
| --- | --- | --- | --- | --- |
| N/A^a^ | | - N/A | - Do not code remarks associated with common practices of group facilitation (unless specifically related to one of the core principles of deliberation). - Do not code prepared didactic presentations or informational or procedural remarks | (1) calling on persons (unless soliciting persons who have been quiet), (2) assuring deliberants that this is a safe space (unless stressing the need for diverse opinions), (3) stating that everyone’s ideas are valued (as mentioned earlier), (4) acknowledging deliberants’ responses (“that’s a good point”), (5) summarizing and reflecting on what individual deliberants say, and (6) asking deliberants to clarify or expand on what they have said. |
| **Coding for remarks consistent with the core principles** | | | | |
|  | EP^b^ | - Do code each remark that invites a specific deliberant’s participation if that deliberant has been quiet or not weighed in on a topic - Do code each remark that invites a subgroup’s (eg, young persons, persons who have been quiet) participation if that subgroup has been quiet or not weighed in on a topic | - Do not code common practices of group facilitation as described above that encourage participation but do not address equal participation | X (who has not yet spoken), would you care to weigh in on this topic? We would value your ideas. |
|  | RO^c^ | - Do code each remark that encourages deliberants to be respectful to others - Do code each remark that encourages deliberants to listen to the diverse opinions of others | - Do not code remarks in which team members show (model) respect for others - Do not code remarks in which team members encourage deliberants to express diverse opinions (code them under expression of diverse opinions) | A basic principle of deliberation is that everyone respects the opinions of others even if they differ from our own. |
|  | SP^d^ | - Do code each remark that encourages deliberants to consider what is best for society or the civic good rather than what is best for them or their families personally or acknowledges when they do so | - Do not code remarks that encourage deliberants to focus on the good of the deliberant group | One of the goals of the deliberation is to find solutions that will promote the common good of society. |
|  | RJ^e^ | - Do code each remark that encourages deliberants to draw on information learned at the deliberation or acknowledges when they do so - Do code each remark that encourages deliberants to draw on facts or credible information obtained outside the deliberations or acknowledges when they do so - Do code each remark that encourages deliberants to make logical or coherent arguments or acknowledges when they do so | - Do not code remarks that encourage deliberants to consider the opinions of others (code them under compromise or consensus) - Do not code remarks that provide didactic information | What information have you heard that most affects your opinion? |
|  | ED^f^ | - Do code each remark that encourages deliberants to express disagreements or varying points of view or acknowledges when they do so - Do code each remark that encourages deliberants to express what others with diverse opinions might contribute to the discussion, although they are not present | - Do not code remarks that encourage deliberants to listen to diverse opinions (code them under respect for others) - Do not code general remarks that encourage deliberants to express their opinions (must encourage them to express differing opinions) | X and Y are saying that they believe xxxx. Does anyone have a different opinion? |
|  | CC^g^ | - Do code each remark that encourages deliberants to consider the points of view expressed by other deliberants to move toward consensus or acknowledges when they do so - Do code each remark that encourages deliberants to work toward finding a common ground or acknowledges when they do so - Do code remarks that discuss the difference between agreement and consensus - Do code remarks that introduce the idea of trade-offs | - Do not code remarks that summarize what the deliberants have seemed to agree upon | You have expressed a variety of diverse ideas. In all of those ideas, what do you see as common ground? |
| **Remarks inconsistent with the core principles** | | | | |
|  | EP | - Do code each remark that engages certain deliberants or subgroups to the exclusion of others, such as by calling on members who have already spoken often instead of those who have not yet spoken | - Do not code remarks in which team members call on participants who indicate a wish to speak (ie, raise their hand) even if they have spoken often | X (a frequent speaker), you have expressed a great opinion. Would you say more? |
|  | RO | - Do code each remark that fails to acknowledge disrespect among deliberants | - N/A | (After a disrespectful comment) Let’s move on now. |
|  | SP | - Do code each remark that encourages deliberants to focus on what is best for them or their families rather than what is best for society or the civic good | - Do not code remarks in which team members acknowledge personal experiences, values, or feelings expressed by deliberants | What decision would work best in your family? |
|  | RJ | - Do code each remark that encourages deliberants to call on their own feelings or personal experiences rather than information shared at the deliberation to justify their opinions | - Do not code remarks in which team members acknowledge personal experiences, values, or feelings expressed by deliberants | How would you feel if your child participated in a study that you did not approve of? |
|  | ED | - Do code each remark that discourages deliberants from discussing points of view that differ from those of other deliberants or from those of the research team members - Do code team member remarks that reflect their own opinions rather than elicit the participation of deliberants | - Do not code remarks in which team members provide clarification of misinformation | X and Y have agreed on xxxx. I think that is a great point. Does anyone else feel this way? |
|  | CC | - Do code each remark that encourages participants to stick to their own point of view without considering the views of other deliberants - Do code each remark in which team members summarize what they believe to be the consensus of the group rather than eliciting deliberants’ perceptions of consensus | - Do not code remarks in which team members reflect on deliberants’ statements about what they view as a consensus the group reached - Do not code remarks in which team members summarize participants’ points if they are not interpreting a consensus | Let me summarize what I think you are all saying... |

^a^N/A: not applicable.

^b^EP: equal participation.

^c^RO: respect for others.

^d^SP: adoption of a societal perspective.

^e^RJ: reasoned justification of ideas.

^f^ED: expression of diverse opinions.

^g^CC: compromise or movement toward consensus.
