# Supplementary material for: Assessing Facilitator Fidelity to Principles of Public Deliberation: Tutorial
Source: JMIR Form Res. 2023 Dec 13;7:e51202. doi: 10.2196/51202 (PMC10753414; doi:10.2196/51202)
Supplement: Multimedia Appendix 2 [file formative_v7i1e51202_app2.docx]

**Table S2.** Code display table example.

| Deliberation principle, facilitator, and final code | | | | Session 1 plenary | Breakout 1.1 | Breakout 1.2 | Session 2 plenary | Breakout 2.1 | Breakout 2.2 | Session 3 plenary | Session 4 plenary |
| --- | --- | --- | --- | --- | --- | --- | --- | --- | --- | --- | --- |
| **Remarks consistent with principles of deliberation** | | | | | | | | | | | |
|  | **EP^a^** | | | | | | | | | | |
|  |  | Plenary facilitator 1 | EP | 74-79 | N/A^b^ | N/A | N/A | N/A | N/A | 565-566 and 793-794 | 458-460 and 1316-1318 |
|  |  | Plenary facilitator 2 | EP | N/A | N/A | N/A | N/A | N/A | N/A | N/A | N/A |
|  |  | Breakout group facilitator 1 | EP | N/A | 695-696 | N/A | N/A | N/A | N/A | N/A | N/A |
|  |  | Breakout group facilitator 2 | EP | N/A | N/A | N/A | N/A | N/A | 318-320 | N/A | N/A |
|  |  | Research team member 1 | EP | N/A | N/A | N/A | N/A | N/A | N/A | N/A | N/A |
|  |  | Research team member 2 | EP | N/A | N/A | N/A | N/A | N/A | 882-883 | 691-693 | N/A |
|  |  | Research team member 3 | EP | N/A | N/A | N/A | N/A | N/A | N/A | N/A | N/A |
|  |  | Research team member 4 | EP | N/A | N/A | N/A | N/A | N/A | N/A | N/A | N/A |
|  |  | Site staff member | EP | N/A | N/A | N/A | N/A | N/A | N/A | N/A | N/A |
|  |  | Expert stakeholder (physician) | EP | N/A | N/A | N/A | N/A | N/A | N/A | N/A | N/A |
|  |  | Expert stakeholder (IRB^c^ administrator) | EP | N/A | N/A | N/A | N/A | N/A | N/A | N/A | N/A |
|  | **RO^d^** | | | | | | | | | | |
|  |  | Plenary facilitator 1 | RO | 68-69, 72-74, and 84-85 | N/A | N/A | 220 | N/A | N/A | N/A | N/A |
|  |  | Plenary facilitator 2 | RO | N/A | N/A | N/A | N/A | N/A | N/A | N/A | N/A |
|  |  | Breakout group facilitator 1 | RO | N/A | N/A | N/A | N/A | 200-202 | N/A | N/A | N/A |
|  |  | Breakout group facilitator 2 | RO | N/A | N/A | N/A | N/A | N/A | 36-37 and 511-513 | N/A | N/A |
|  |  | Research team member 1 | RO | N/A | N/A | N/A | N/A | N/A | N/A | N/A | N/A |
|  |  | Research team member 2 | RO | N/A | N/A | N/A | N/A | N/A | N/A | N/A | N/A |
|  |  | Research team member 3 | RO | N/A | N/A | N/A | N/A | N/A | N/A | N/A | N/A |
|  |  | Research team member 4 | RO | 123-124 | N/A | N/A | N/A | N/A | N/A | N/A | N/A |
|  |  | Site staff member | RO | N/A | N/A | N/A | N/A | N/A | N/A | N/A | N/A |
|  |  | Expert stakeholder (physician) | RO | N/A | N/A | N/A | N/A | N/A | N/A | N/A | N/A |
|  |  | Expert stakeholder (IRB administrator) | RO | N/A | N/A | N/A | N/A | N/A | N/A | N/A | N/A |
|  | **ED**^e^ | | | | | | | | | | |
|  |  | Plenary facilitator 1 | ED | 59-60, 69-72, and 183-190 | N/A | N/A | 107-109, 356, and 450-452 | 1196-1197 | N/A | 95-97, 1419-1420, 1495-1496, and 1576-1577 | 296-298, 1434-1437, and 1769-1771 |
|  |  | Plenary facilitator 2 | ED | N/A | N/A | N/A | N/A | N/A | N/A | N/A | N/A |
|  |  | Breakout group facilitator 1 | ED | N/A | 808-814 | N/A | N/A | 49-51, 51-54, 92-94, 104-110, 110, 120-122, 122-123, 155-156, 177-181, and 417-418 | N/A | N/A | N/A |
|  |  | Breakout group facilitator 2 | ED | N/A | N/A | 859-861 | N/A | N/A | 185-190, 337-339, 383-385, 396-400, 444-448, 487-488, 634-644, and 795-797 | N/A | N/A |
|  |  | Research team member 1 | ED | N/A | N/A | N/A | N/A | N/A | N/A | 1766-1770 | N/A |
|  |  | Research team member 2 | ED | N/A | N/A | N/A | N/A | N/A | N/A | N/A | 301-304 |
|  |  | Research team member 3 | ED | N/A | N/A | N/A | N/A | N/A | N/A | N/A | N/A |
|  |  | Research team member 4 | ED | N/A | N/A | N/A | N/A | N/A | N/A | N/A | N/A |
|  |  | Site staff member | ED | N/A | N/A | N/A | N/A | N/A | N/A | N/A | N/A |
|  |  | Expert stakeholder (physician) | ED | N/A | N/A | N/A | N/A | N/A | N/A | N/A | N/A |
|  |  | Expert stakeholder (IRB administrator) | ED | N/A | N/A | N/A | N/A | N/A | N/A | N/A | N/A |
|  | **SP^f^** | | | | | | | | | | |
|  |  | Plenary facilitator 1 | SP | 43-47 | N/A | N/A | N/A | N/A | N/A | N/A | N/A |
|  |  | Plenary facilitator 2 | SP | N/A | N/A | N/A | N/A | N/A | N/A | N/A | N/A |
|  |  | Breakout group facilitator 1 | SP | N/A | N/A | N/A | N/A | N/A | N/A | N/A | N/A |
|  |  | Breakout group facilitator 2 | SP | N/A | N/A | N/A | N/A | N/A | 642-644 | N/A | N/A |
|  |  | Research team member 1 | SP | N/A | N/A | N/A | N/A | 574-576 | N/A | N/A | N/A |
|  |  | Research team member 2 | SP | N/A | N/A | 544-547 | N/A | N/A | N/A | N/A | N/A |
|  |  | Research team member 3 | SP | N/A | N/A | N/A | N/A | N/A | N/A | N/A | N/A |
|  |  | Research team member 4 | SP | N/A | N/A | N/A | N/A | N/A | N/A | N/A | N/A |
|  |  | Site staff member | SP | N/A | N/A | N/A | N/A | N/A | N/A | N/A | N/A |
|  |  | Expert stakeholder (physician) | SP | N/A | N/A | N/A | N/A | N/A | N/A | N/A | N/A |
|  |  | Expert stakeholder (IRB administrator) | SP | N/A | N/A | N/A | N/A | N/A | N/A | N/A | N/A |
|  | **RJ^g^** | | | | | | | | | | |
|  |  | Plenary facilitator 1 | RJ | N/A | N/A | N/A | N/A | N/A | N/A | 645-653 | 603-606 and1426-1427 |
|  |  | Plenary facilitator 2 | RJ | N/A | N/A | N/A | N/A | N/A | N/A | N/A | N/A |
|  |  | Breakout group facilitator 1 | RJ | N/A | N/A | N/A | N/A | N/A | N/A | N/A | N/A |
|  |  | Breakout group Facilitator 2 | RJ | N/A | N/A | 550-553 | N/A | N/A | N/A | N/A | N/A |
|  |  | Research team member 1 | RJ | N/A | N/A | N/A | N/A | N/A | N/A | N/A | N/A |
|  |  | Research team member 2 | RJ | N/A | N/A | N/A | N/A | N/A | 979-982 | 392 | N/A |
|  |  | Research team member 3 | RJ | N/A | N/A | N/A | N/A | N/A | N/A | N/A | N/A |
|  |  | Research team member 4 | RJ | N/A | N/A | N/A | N/A | N/A | N/A | N/A | N/A |
|  |  | Site staff member | RJ | N/A | N/A | N/A | N/A | N/A | N/A | N/A | N/A |
|  |  | Expert stakeholder (physician) | RJ | N/A | N/A | N/A | N/A | 169-172 | N/A | N/A | N/A |
|  |  | Expert stakeholder (IRB administrator) | RJ | N/A | N/A | N/A | N/A | N/A | N/A | N/A | N/A |
|  | **CC^h^** | | | | | | | | | | |
|  |  | Plenary facilitator 1 | CC | 61, 61-63, 81-84, 182-183, 190-194, 196-198, 199-202, 203-206, and 206-209 | 994-996 | N/A | 100-102, 109-110, 220-221, 446-450, and 450-452 | 1185-1198, 1192-1194, and 1200 | N/A | 35-38 | 170-173, 181-184, 184-189, 192-194, 593-594, 1303-1307, 1422-1426, 1433-1434, and 1450-1452 |
|  |  | Plenary facilitator 2 | CC | N/A | N/A | N/A | N/A | N/A | N/A | N/A | N/A |
|  |  | Breakout group facilitator 1 | CC | N/A | N/A | N/A | N/A | N/A | N/A | N/A | N/A |
|  |  | Breakout group facilitator 2 | CC | N/A | N/A | N/A | N/A | N/A | N/A | N/A | N/A |
|  |  | Research team member 1 | CC | N/A | N/A | N/A | N/A | N/A | N/A | N/A | N/A |
|  |  | Research team member 2 | CC | N/A | N/A | N/A | N/A | N/A | N/A | N/A | N/A |
|  |  | Research team member 3 | CC | N/A | N/A | N/A | N/A | N/A | N/A | N/A | N/A |
|  |  | Research team member 4 | CC | 124-125 | N/A | N/A | N/A | N/A | N/A | N/A | N/A |
|  |  | Site staff member | CC | N/A | N/A | N/A | N/A | N/A | N/A | N/A | N/A |
|  |  | Expert stakeholder (physician) | CC | N/A | N/A | N/A | N/A | N/A | N/A | N/A | N/A |
|  |  | Expert stakeholder (IRB administrator) | CC | N/A | N/A | N/A | N/A | N/A | N/A | N/A | N/A |
| **Remarks inconsistent the with principles of deliberation** | | | | | | | | | | | |
|  | **EP** | | | | | | | | | | |
|  |  | Plenary facilitator 1 | EP^i^ | N/A | N/A | N/A | N/A | N/A | N/A | 1422-1423 | 687-690 |
|  |  | Plenary facilitator 2 | EP^i^ | N/A | N/A | N/A | N/A | N/A | N/A | N/A | N/A |
|  |  | Breakout group facilitator 1 | EP^i^ | N/A | N/A | N/A | N/A | N/A | N/A | N/A | N/A |
|  |  | Breakout group facilitator 2 | EP^i^ | N/A | N/A | N/A | N/A | N/A | N/A | N/A | N/A |
|  |  | Research team member 1 | EP^i^ | N/A | N/A | N/A | N/A | N/A | N/A | N/A | N/A |
|  |  | Research team member 2 | EP^i^ | N/A | N/A | N/A | N/A | N/A | N/A | N/A | N/A |
|  |  | Research team member 3 | EP^i^ | N/A | N/A | N/A | N/A | N/A | N/A | N/A | N/A |
|  |  | Research team member 4 | EP^i^ | N/A | N/A | N/A | N/A | N/A | N/A | N/A | N/A |
|  |  | Site staff member | EP^i^ | N/A | N/A | N/A | N/A | N/A | N/A | N/A | N/A |
|  |  | Expert stakeholder (physician) | EP^i^ | N/A | N/A | N/A | N/A | N/A | N/A | N/A | N/A |
|  |  | Expert stakeholder (IRB administrator) | EP^i^ | N/A | N/A | N/A | N/A | N/A | N/A | N/A | N/A |
|  | **RO** | | | | | | | | | | |
|  |  | Plenary facilitator 1 | RO^i^ | N/A | N/A | N/A | N/A | N/A | N/A | N/A | N/A |
|  |  | Plenary facilitator 2 | RO^i^ | N/A | N/A | N/A | N/A | N/A | N/A | N/A | N/A |
|  |  | Breakout group Facilitator 1 | RO^i^ | N/A | N/A | N/A | N/A | N/A | N/A | N/A | N/A |
|  |  | Breakout group Facilitator 2 | RO^i^ | N/A | N/A | N/A | N/A | N/A | N/A | N/A | N/A |
|  |  | Research team member 1 | RO^i^ | N/A | N/A | N/A | N/A | N/A | N/A | N/A | N/A |
|  |  | Research team member 2 | RO^i^ | N/A | N/A | N/A | N/A | N/A | N/A | N/A | N/A |
|  |  | Research team member 3 | RO^i^ | N/A | N/A | N/A | N/A | N/A | N/A | N/A | N/A |
|  |  | Research team member 4 | RO^i^ | N/A | N/A | N/A | N/A | N/A | N/A | N/A | N/A |
|  |  | Site staff member | RO^i^ | N/A | N/A | N/A | N/A | N/A | N/A | N/A | N/A |
|  |  | Expert stakeholder (physician) | RO^i^ | N/A | N/A | N/A | N/A | N/A | N/A | N/A | N/A |
|  |  | Expert stakeholder (IRB administrator) | RO^i^ | N/A | N/A | N/A | N/A | N/A | N/A | N/A | N/A |
|  | **ED** | | | | | | | | | | |
|  |  | Plenary facilitator 1 | ED^i^ | N/A | N/A | N/A | N/A | N/A | N/A | N/A | N/A |
|  |  | Plenary facilitator 2 | ED^i^ | N/A | N/A | N/A | N/A | N/A | N/A | N/A | N/A |
|  |  | Breakout group facilitator 1 | ED^i^ | N/A | N/A | N/A | N/A | N/A | N/A | N/A | N/A |
|  |  | Breakout group facilitator 2 | ED^i^ | N/A | N/A | N/A | N/A | N/A | 677-678 | N/A | N/A |
|  |  | Research team member 1 | ED^i^ | N/A | N/A | N/A | N/A | N/A | N/A | N/A | N/A |
|  |  | Research team member 2 | ED^i^ | N/A | N/A | N/A | N/A | N/A | N/A | N/A | 1945-1949 |
|  |  | Research team member 3 | ED^i^ | N/A | N/A | N/A | N/A | N/A | N/A | N/A | N/A |
|  |  | Research team member 4 | ED^i^ | N/A | N/A | N/A | N/A | N/A | N/A | N/A | N/A |
|  |  | Site staff member | ED^i^ | N/A | N/A | N/A | N/A | N/A | N/A | N/A | N/A |
|  |  | Expert stakeholder (physician) | ED^i^ | N/A | N/A | N/A | N/A | 498-502 and 543-548 | N/A | N/A | 1290-1293 |
|  |  | Expert stakeholder (IRB administrator) | ED^i^ | N/A | N/A | N/A | N/A | N/A | N/A | N/A | N/A |
|  | **SP** | | | | | | | | | | |
|  |  | Plenary facilitator 1 | SP^i^ | N/A | N/A | N/A | N/A | N/A | N/A | N/A | N/A |
|  |  | Plenary facilitator 2 | SP^i^ | N/A | N/A | N/A | N/A | N/A | N/A | N/A | N/A |
|  |  | Breakout group facilitator 1 | SP^i^ | N/A | 564-566 | N/A | N/A | N/A | N/A | N/A | N/A |
|  |  | Breakout group facilitator 2 | SP^i^ | N/A | N/A | 580-582 | N/A | N/A | N/A | N/A | N/A |
|  |  | Research team member 1 | SP^i^ | N/A | N/A | N/A | N/A | N/A | N/A | N/A | N/A |
|  |  | Research team member 2 | SP^i^ | N/A | N/A | N/A | N/A | N/A | N/A | N/A | N/A |
|  |  | Research team member 3 | SP^i^ | N/A | N/A | N/A | N/A | N/A | N/A | N/A | N/A |
|  |  | Research team member 4 | SP^i^ | N/A | N/A | N/A | N/A | N/A | N/A | N/A | N/A |
|  |  | Site staff member | SP^i^ | N/A | N/A | N/A | N/A | N/A | N/A | N/A | N/A |
|  |  | Expert stakeholder (physician) | SP^i^ | N/A | N/A | N/A | N/A | N/A | N/A | N/A | N/A |
|  |  | Expert stakeholder (IRB administrator) | SP^i^ | N/A | N/A | N/A | N/A | N/A | N/A | N/A | N/A |
|  | **RJ** | | | | | | | | | | |
|  |  | Plenary facilitator 1 | RJ^i^ | N/A | N/A | N/A | N/A | N/A | N/A | N/A | N/A |
|  |  | Plenary facilitator 2 | RJ^i^ | N/A | N/A | N/A | N/A | N/A | N/A | N/A | N/A |
|  |  | Breakout group facilitator 1 | RJ^i^ | N/A | N/A | N/A | N/A | N/A | N/A | N/A | N/A |
|  |  | Breakout group facilitator 2 | RJ^i^ | N/A | N/A | N/A | N/A | N/A | N/A | N/A | N/A |
|  |  | Research team member 1 | RJ^i^ | N/A | N/A | N/A | N/A | N/A | N/A | N/A | N/A |
|  |  | Research team member 2 | RJ^i^ | N/A | N/A | N/A | N/A | N/A | N/A | N/A | N/A |
|  |  | Research team member 3 | RJ^i^ | N/A | N/A | N/A | N/A | N/A | N/A | N/A | N/A |
|  |  | Research team member 4 | RJ^i^ | N/A | N/A | N/A | N/A | N/A | N/A | N/A | N/A |
|  |  | Site staff member | RJ^i^ | N/A | N/A | N/A | N/A | N/A | N/A | N/A | N/A |
|  |  | Expert stakeholder (physician) | RJ^i^ | N/A | N/A | N/A | N/A | N/A | N/A | N/A | N/A |
|  |  | Expert stakeholder (IRB administrator) | RJ^i^ | N/A | N/A | N/A | N/A | N/A | N/A | N/A | N/A |
|  | **CC** | | | | | | | | | | |
|  |  | Plenary facilitator 1 | CC^i^ | N/A | N/A | N/A | 346-356 and 437-446 | N/A | N/A | N/A | 279-286, 1507-1511, and 1617-1624 |
|  |  | Plenary facilitator 2 | CC^i^ | N/A | N/A | N/A | N/A | N/A | N/A | N/A | N/A |
|  |  | Breakout group facilitator 1 | CC^i^ | N/A | N/A | N/A | N/A | 107-110 | N/A | N/A | 1568-1576 |
|  |  | Breakout group facilitator 2 | CC^i^ | N/A | N/A | N/A | N/A | N/A | N/A | N/A | N/A |
|  |  | Research team member 1 | CC^i^ | N/A | N/A | N/A | 376-405 | N/A | N/A | N/A | N/A |
|  |  | Research team member 2 | CC^i^ | N/A | N/A | N/A | N/A | N/A | N/A | N/A | N/A |
|  |  | Research team member 3 | CC^i^ | N/A | N/A | N/A | N/A | N/A | N/A | N/A | N/A |
|  |  | Research team member 4 | CC^i^ | N/A | N/A | N/A | N/A | N/A | N/A | N/A | N/A |
|  |  | Site staff member | CC^i^ | N/A | N/A | N/A | N/A | N/A | N/A | N/A | N/A |
|  |  | Expert stakeholder (physician) | CC^i^ | N/A | N/A | N/A | N/A | N/A | N/A | N/A | 1551-1553 |
|  |  | Expert stakeholder (IRB administrator) | CC^i^ | N/A | N/A | N/A | N/A | N/A | N/A | N/A | N/A |

^a^EP: equal participation.

^b^N/A: not applicable (eg, facilitator 1 was not part of breakout 1.1; facilitator was not present).

^c^IRB: institutional review board.

^d^RO: respect for others.

^e^ED: expression of diverse opinions.

^f^SP: adoption of a societal perspective.

^g^RJ: reasoned justification of ideas.

^h^CC: compromise or movement toward consensus.

^i^Remark was relevant to but inconsistent with the principle.
